# Adenomyotic Lesions Are Induced in the Mouse Uterus after Exposure to NSAID and EE2 Mixtures at Environmental Doses

**DOI:** 10.3390/ijms25042003

**Published:** 2024-02-07

**Authors:** Brigitte Boizet-Bonhoure, Stéphanie Déjardin, Mélissa Girard, Quentin Durix, Francis Poulat, Pascal Philibert

**Affiliations:** 1 Développement et Pathologie de la Gonade, Institut de Génétique Humaine, Centre National de la Recherche Scientifique, Université de Montpellier UMR9002, 34090 Montpellier, France; stephanie.dejardin@igh.cnrs.fr (S.D.); melissa080598@gmail.com (M.G.); francis.poulat@igh.cnrs.fr (F.P.); 2 IExplore-RAM, Institut de Génomique Fonctionnelle, Centre National de la Recherche Scientifique, INSERM, Université de Montpellier, 34090 Montpellier, France; quentin.durix@igf.cnrs.fr; 3 Laboratoire de Biochimie et Biologie Moléculaire, Hôpital Carémeau, CHU de Nîmes, 30029 Nîmes, France

**Keywords:** NSAIDs, ethinylestradiol, uterus, adenomyosis, fibrosis, intergenerational inheritance

## Abstract

The aim of this study was to assess the long-term effect of exposure to environmentally relevant doses of non-steroidal anti-inflammatory drugs (NSAIDs; ibuprofen, and diclofenac) and 17β-ethinylestradiol (EE2) on the mouse uterus. NSAID-EE2 mixtures were administered in the drinking water from gestational day 8 until 8 weeks post-birth (i.e., during embryo development, lactation, puberty, and sexual maturity). The incidence of adenomyosis lesions (presence of endometrial glands in the inner myometrium) increased up to 60% in the uterus of 8-week-old exposed females (F1) and to 85% in F2 females (exposed father). Histological analysis revealed aberrant proliferation and apoptosis, vacuolization of epithelial cells, and increased incidence of abnormal glands in the luminal and glandular epithelium in F1 and F2 uteri. Moreover, myofibroblast proportion (alpha-smooth muscle actin (α-SMA) expression analysis) and collagen expression (Picrosirius red stain; a fibrosis hallmark) were increased in F1 and F2 endometrium. Connexin-43 was aberrantly distributed in the endometrial stroma and glands of F1 and F2 uteri. Conversely, uterine 17β-estradiol and progesterone levels were not affected in F1 and F2 females. These findings demonstrated that in mice, chronic exposure to NSAID and EE2 mixtures at environmental doses intergenerationally affects uterine physiology, particularly the endometrium. It may serve as a model to study the pathophysiology of human adenomyosis.

## 1. Introduction

Adenomyosis and endometriosis are gynecological disorders that affect millions of women worldwide [1,2]. Adenomyosis is defined by the presence of ectopic endometrial glands and mesenchymal stroma in the uterine myometrium [3], whereas endometriosis is characterized by the presence of endometrial glands and stroma at extrauterine sites. Their etiology remains uncertain; however, both have similar symptoms (pelvic pain, heavy menstrual bleeding, and dysmenorrhea) and contribute to fertility disorders [4]. Moreover, adenomyosis often coexists with other gynecological disorders and uterine fibroids (uterine leiomyoma) and is often associated with endometrial carcinoma. Adenomyosis and endometriosis are chronic estrogen-dependent inflammatory diseases [5], suggesting a common or overlapping natural history [6]. The pathogenesis of adenomyosis includes tissue injury and repair mechanisms [7], leading to abnormal functions of the junctional zone at the endometrium-myometrial interface [8]. Repeated tissue injury and repair leads to increased fibrosis in the endometrium, which also plays a role in adenomyosis occurrence and pathogenesis [9]. 

Human and animal studies have identified various growth factors, inflammatory molecules, angiogenic factors, and extracellular matrix (ECM) remodeling proteins as key mediators of these disorders [3]. In the uterus, the collagen ECM plays a dynamic structural and functional role in tissue remodeling of the cycling uterus and during pregnancy [10]. Deregulation of ECM function and collagen accumulation contributes to endometrial adenocarcinoma, fibroids (leiomyomas), and endometriosis [11,12]. Furthermore, gap junction family and connexins have essential roles in the physiology of female reproductive organs. Particularly, connexin 43 (Cx43) plays a significant role in cell–cell communication during implantation and decidualization [13]. Aberrant epithelial distribution of Cx43 was reported in ectopic endometriotic glands of adenomyosis lesions in cows [14].

To study adenomyosis pathophysiology and identify adenomyosis biomarkers, murine models of adenomyosis have been developed through exposure of newborn mice to tamoxifen [15,16,17,18], progesterone [19] or estradiol [20,21,22]. Similarly, several genetic models of adenomyosis have been identified. Indeed, constitutive activation of β-catenin [23], follicle-stimulating hormone receptor haploinsufficiency [24], overexpression of human estrogen biosynthetic enzyme 1 in transgenic mice [25] and knock-out of the *L-Pgds* and *H-Pgds* genes that encode lipocalin- and hematopoietic-type prostaglandin D2 synthases [26] promote adenomyosis development. 

Exposure to environmental endocrine-disrupting chemicals (EDCs) can lead to adverse reproductive outcomes [27], and some EDCs target the uterus, suggesting a putative role in adenomyosis [28,29,30]. The incidence of uterine structural changes, abnormal pregnancies, and decreased fertility is higher in women who were exposed in utero to diethylstilbestrol (DES) [31]. Epidemiology studies suggested that exposure to dioxin [32] or polychlorinated biphenyls [33] might promote adenomyosis development in women. In rodents, exposure to dioxin [34], DES [19], bisphenol A (BPA) [35,36], 17β-ethinylestradiol (EE2), a synthetic xenoestrogen [37,38,39], and tributyltin, an environmental androgen [40], has been associated with pyometra, disorganization of the glandular, luminal epithelium and endometrial compartments, dysplasia, fibrosis, and adenomyosis. Moreover, exposure to phthalates and their metabolites leads to transgenerational effects on the uterine morphology, including the altered proliferation of the luminal epithelium, abnormally large and dilated endometrial glands, and signs of adenomyosis (endometrial glands surrounded by elevated α smooth muscle actin, α-SMA, expression) [41,42].

Non-steroidal anti-inflammatory drugs (NSAIDs), such as ibuprofen (IBU) and diclofenac (DCF), are over-the-counter drugs commonly prescribed to treat inflammation and pain [43], and the xenoestrogen EE2 is widely used in combination with progestins for contraception and for hormone replacement therapy [44]. Many epidemiological studies on in utero exposure to therapeutic doses of aspirin, IBU, or acetaminophen during the first months of gestation [43,45] and ex vivo/in vivo studies in rodent models [46] showed that these molecules are EDCs. EE2 and other estrogenic derivatives have been identified as EDCs that may affect early development and fertility in exposed rodents and their offspring [47,48] and reproductive health in humans [49]. 

These drugs and 2-hydroxy-ibuprofen (2h-IBU, an IBU metabolite) are among the most present molecules at low concentrations in drinking water [50,51] as a result of their incomplete removal by wastewater treatment plans. Recently, we showed that chronic exposure to a mixture containing IBU, 2h-IBU, DCF, and EE2 at two environmentally relevant doses, intergenerationally impairs ovary development and physiology in F1 mice and their offspring. This leads to early puberty onset in F1-exposed postnatal females and their F2 offspring [52] and altered reproductive organ maturation in adult F1 and F2 females [53]. F1 and F2 females also showed irregular estrous cyclicity, although 17β-estradiol (E2) concentration in serum was not significantly affected [53]. Consequently, fertility (number of pups per litter and number of days between litters) decreased with age in F1 females (exposed) and also in their F2 offspring [53]. However, the effects of these chemicals on the uterus morphology/physiology remain unclear. 

In the present study, we extended our previous work to assess the effects of long-term exposure to IBU, 2h-IBU, DCF, and EE2 mixtures on the uterus morphology and physiology of exposed F1 mice and of their F2 offspring. 

## 2. Results 

### 2.1. Exposure to NSAID and EE2 Mixtures Induces Adenomyosis Lesions in the Mouse Uterus

Exposure to the NSAID and EE2 cocktail at both environmental doses (D1 and D2) did not produce any sign of embryotoxicity (i.e., all pregnant dams successfully delivered their pups, and the number of live pups per litter was similar among the groups) and did not alter the gestation length. No differences in the sex ratio and no gross malformation were observed in pups at delivery or weaning. No significant difference in 8-week-old female body weight and gross genital tract morphology was identified, and no macroscopic uterus abnormality was observed. 

**Figure 1:**
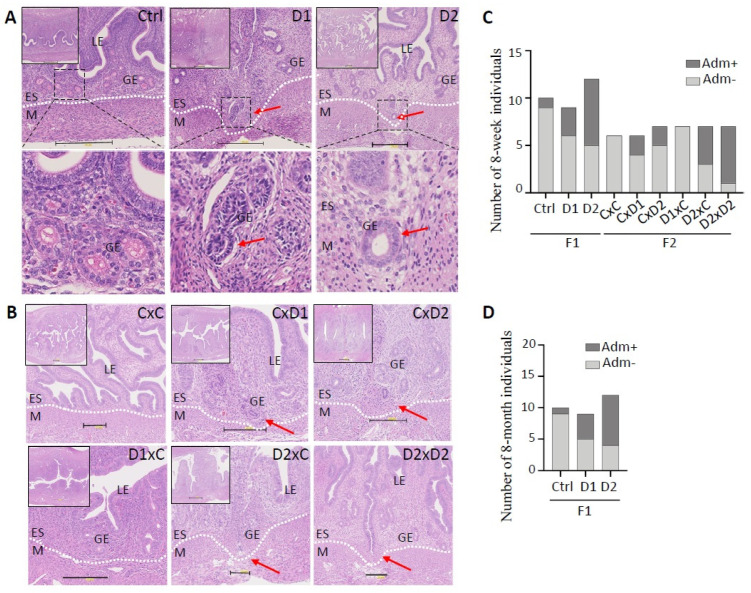
Exposure to the mixture (D1 and D2) promotes adenomyosis development in the uterus of 8-week-old F1 (**A**) and F2 (**B**) females. Representative images of hematoxylin–eosin-stained paraffin-embedded uterus tissue sections. Scale bars: 300 μm (**A**) and 200 μm (**B**). Insets in the upper left corner indicate areas that were enlarged to visualize the luminal (LE) and glandular (GE) epithelium, endometrial stroma (ES), and myometrium (M). The dashed white lines delineated the ES and M compartments. (**A**) The dashed back boxes in the upper panels were enlarged to show endometrial glands (lower panels). Red arrows, glands entering the myometrium. (**C**,**D**) Histograms showing the number of females with (Adm+) and without adenomyosis (Adm−) in 8-week-old F1 and F2 (**C**) and in 8-month-old F1 (**D**) groups. F1 (D1 or D2) males (m) or females (f) were mated with control males (mCtrl) or females (fCtrl) (*n* = 6 groups: mCtrlxfCtrl, mCtrlxfD1, mCtrlxfD2, mD1xfCtrl, mD2xfCtrl, and mD2xfD2) (these mating combinations were simplified to CxC, CxD1, CxD2, D1xC, D2xC, and D2xD2, respectively).

Histological analysis of uteri from 8-week-old F1 (exposed) females (Figure 1A) and their F2 offspring (Figure 1B) after HE staining showed the presence of nascent adenomyosis lesions (i.e., endometrial glands invaginated in the myometrium) in 3/9 (33%) F1-D1 and 7/12 (58%) F1-D2 uteri (Figure 1A,C). Adenomyotic foci were identified in the uterus of 2/6 (33%), 2/7 (28%), 4/7 (57%), and 6/7 (85%) F2 CxD1, F2 CxD2, F2 D2xC, and F2 D2xD2 females, respectively (Figure 1B,C). In control F1 (Ctrl) and F2 (CxC) females, the endometrium and myometrium layers were well demarcated (Figure 1A,B), and adenomyosis lesions were present only in 10% (*n* = 1/10) of F1, but not in F2 samples (Figure 1C). 

In 8-month-old F1 females (exposed until 8-weeks of age), adenomyosis frequency (Figure 1D) was similar to the incidence observed in younger mice (Figure 1C): 4/9 (44%) F1-D1 and 8/12 (66%) F1-D2 females, and 1/10 (10%) controls (*n* = 1/10) (Figure 1D). This suggested that adenomyosis lesions did not regress after the exposure end. 

### 2.2. Abnormal Histological Features in the Uterus of F1 and F2 Females

**Figure 2:**
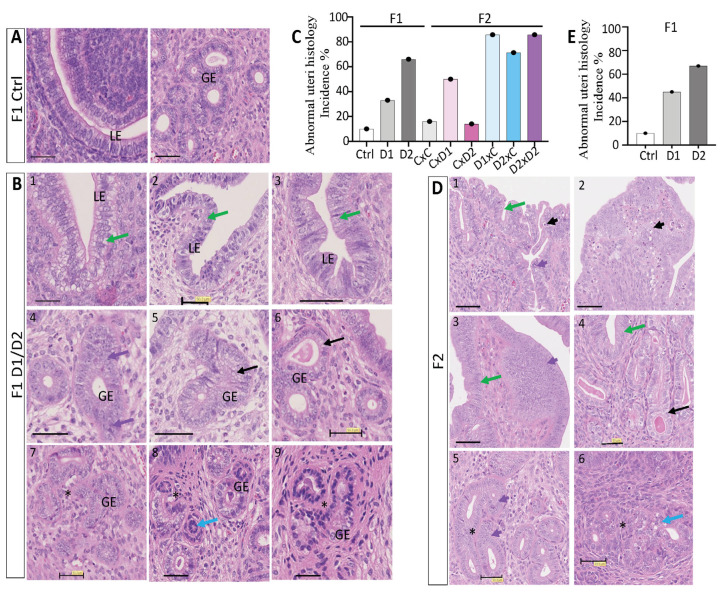
Abnormal histological features in the uterus of 8-week-old F1 and F2 females. Representative images of hematoxylin–eosin-stained paraffin-embedded uterus tissue sections of F1 control (**A**), F1 (**B**), and F2 (**D**) females. Scale bars: 50 μm. The following abnormal features were observed: clear cytoplasm, central nuclei, vacuolization, epithelial thickening (green arrows); dilated glands (black arrows); abnormal glands (blue arrows); proliferating glandular epithelium (GE) cells (purple arrows); hyperplasic luminal epithelium (LE)/GE (short purple arrows); apoptotic LE/GE cells (short black arrows) and gland nest formation (black stars). (**C**,**E**) Histograms showing the incidence (%) of abnormal features in the uterus of 8-week-old F1 and F2 (**C**) and in 8-month-old F1 (**E**) females.

Besides adenomyosis lesions, other morphological abnormalities were more frequently observed in the uterus of exposed F1 and F2 females compared with controls (Figure 2). This included morphological changes in the luminal epithelium, endometrium and/or endometrial glands (Figure 2B,D) in 33% of F1-D1 (*n* = 3/9), 66% of F1-D2 (*n* = 8/12), 50% of F2 CxD1 (*n* = 3/6), 14% of F2 CxD2 (*n* = 1/7), 85% of F2 D1xC (*n* = 6/7), 71% of F2 D2xC (*n* = 5/7), and 85% of F2 D2xD2 (*n* = 6/7) females (Figure 2C). These data suggested an increased incidence of uterine abnormalities in F2 females with an exposed father (Figure 2C).

Exposure increased the incidence of multilayered luminal epithelium in the F1-D1 and F1-D2 (Figure 2B1–3) and F2 (Figure 2D1–4) groups (Figure 2C), compared with the single layer of simple columnar epithelium in controls [54] (Figure 2A). Abnormal epithelial cells with clear cytoplasm or vacuolization, with atypical arrangement of the nuclei also being observed (Figure 2B1–3,D3,4, green arrows). Furthermore, hyperplasic luminal epithelium with elongated nuclei was present (Figure 2D, short purple arrows), with apoptotic luminal or endometrial cells in F2 D1xC, D2xC and D2xD2 females (Figure 2D1,2, short black arrows). Different morphological types of endometrial glands were observed: proliferative/hyperplasic (Figure 2B4,D5, purple arrows; Figure 2D5, short purple arrow) or abnormal glandular epithelium (Figure 2B8, blue arrow), dilated endometrial glands with enlarged epithelium instead of the normal cuboid epithelium [55] (Figure 2B5,6,D4, black arrows), and gland nests with daughter glands that formed conglomerates (Figure 2B7–9,D5,6, black stars). However, exposure to the NSAID and EE2 mixture did not alter the number of glands and the inner or outer myometrium thickness in the uterus of F1 and F2 females. In the uterus of 8-month-old F1 females (Figure 2E), the incidence of abnormal features was similar to what observed in 8-week-old females (Figure 2C). 

### 2.3. Hormone Dosages in the Uterus of F1 and F2 Females

**Figure 3:**
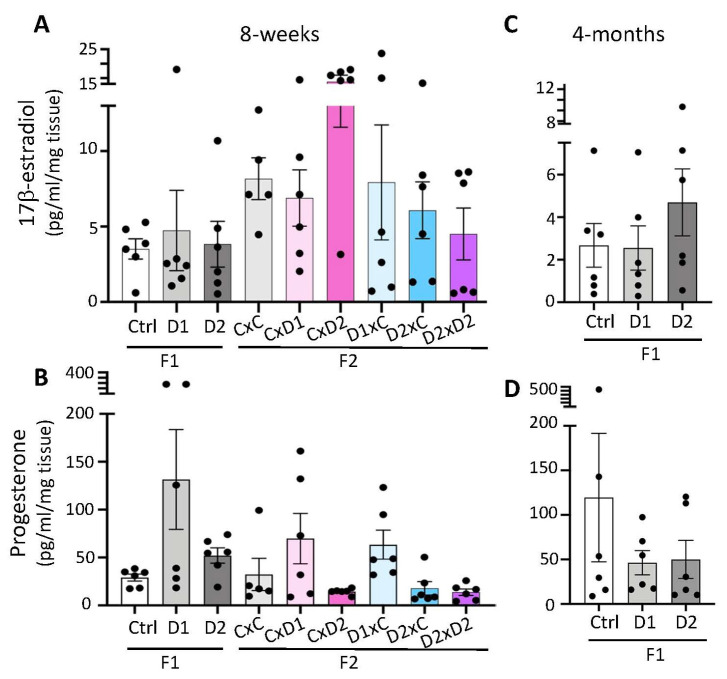
Steroid hormone production in 8-week-old F1 and F2 (**A**,**B**) and 4-month-old F1 (**C**,**D**) uteri. The 17β-estradiol (**A**,**C**) and progesterone (**B**,**D**) secretion were measured in uterine extracts by ELISA. Data are the mean ± SEM and are expressed in pg/mL/mg tissue.

In 8-week-old females, steroid quantification showed that uterine E2 and P4 levels were not significantly different in F1 or F2 females and their respective controls (*p* > 0.05) (Figure 3A,B). However, the mean E2 concentration in the uterus of F2 CxD2 females tended to be higher (13.8 pg/mL) than in controls (8.1 ng/mL) and the mean P4 concentration tended to be higher in F1-D1 (131.5 pg/mL) and F1-D2 (55 pg/mL), in F2 CxD1 and F2 D1xC (69.9–63.5 pg/mL) females and tended to be lower in F2 CxD2, F2 D2xC and F2 D2xD2 (15 pg/mL) females than in controls (30 pg/mL). Similarly, in 4-month-old F1 animals, the mean E2 concentration in the uterus tended to be higher in F1-D2 (4.7 pg/mL) than in control females (2.6 ng/mL) (Figure 3C), and the mean P4 concentration tended to be lower in F1-D1 (46.3 pg/mL) and F1 D2 (50 pg/mL) than in control females (119.5 pg/mL) (Figure 3D). 

### 2.4. Abnormal Smooth Muscle Actin and Collagen Deposition in the Endometrium of F1 and F2 Females

**Figure 4:**
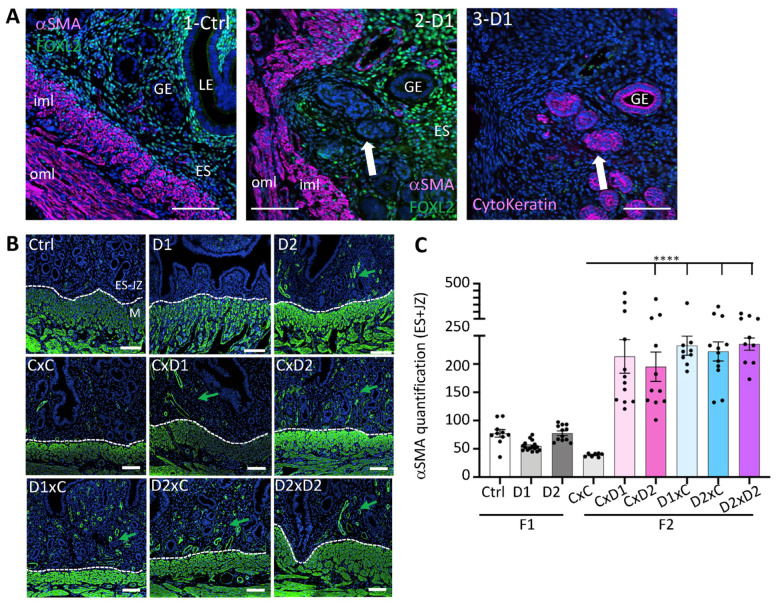
α-SMA expression in F1 and F2 uteri. (**A**) Representative immunofluorescence of α-SMA, FOXL2, and pan-cytokeratin expression in control uteri (1-Ctrl), and adenomyosis lesions in F1-D1 uteri (2-D1, 3-D1). LE: luminal epithelium; GE: glandular epithelium; ES: endometrial stroma; iml: inner myometrium layer; oml: outer myometrium layer. White arrow, adenomyosis lesion. (**B**) Representative immunofluorescence images of α-SMA expression (in green) in the uterus of 8-week-old F1 and F2 females; blue, nuclei stained with Hoechst dye. Scale bars: 100 μm (**A**,**B**). Green arrows, α-SMA expression in the endometrial stroma (ES)-junctional zone (JZ) compartments near the myometrium (M); The dashed white lines delineated the ES and M compartments. (**C**) Quantification of α-SMA staining in the ES-JZ compartments using the OMERO software; *n* = 2–3 sections per uterus, *n* = 4–7 per group (*n* = 4–12 regions of interest at the ES-JZ of a constant surface (30 mm^2^)). Data are the mean ± SEM, **** *p* < 0.001.

IF with an antibody against α-SMA (a myofibroblast and smooth muscle marker) showed a strong α-SMA signal in the outer (oml) and inner (iml) myometrium layers in both control and D1 uteri (Figure 4A1,2). The presence of glandular epithelial cells in adenomyotic lesions in the uterus of 8-week-old F1 females was confirmed by IF analysis of cytokeratin expression (an epithelial marker) using a pan-cytokeratin antibody (Figure 4A3). Adenomyotic lesions containing pan-cytokeratin-positive glands were surrounded by cells that strongly expressed α-SMA (Figure 4A2). Quantification of α-SMA staining intensity in the endometrial stroma (ES) and junctional zone (JZ) compartments in the uterus of F1 and F2 females (Figure 4B,C) showed that exposure to this pharmaceutical mixture increased α-SMA expression in the ES and JZ compartments of the uterus from all F2 groups (*p* < 0.0001). This suggested an increased number of myofibroblasts in the uterus of F2 females.

**Figure 5:**
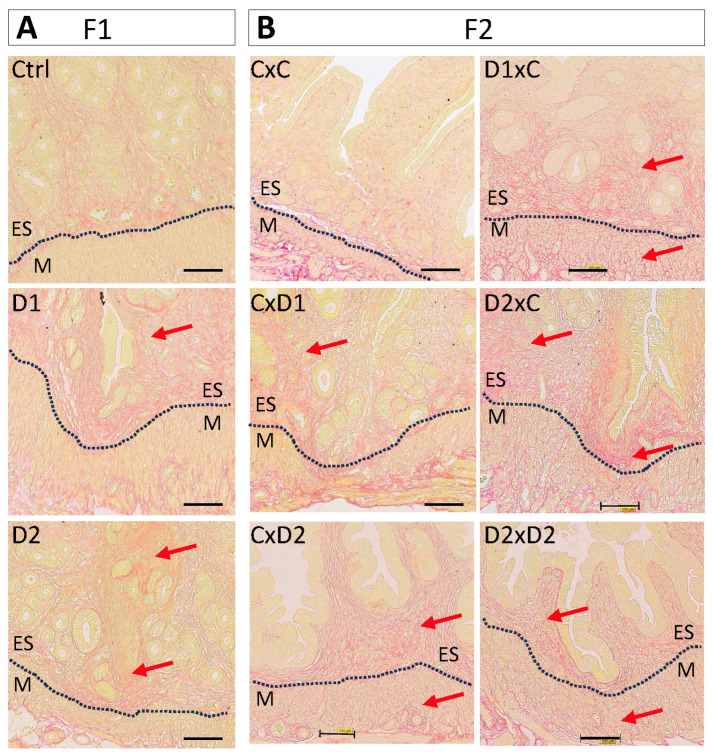
Representative images of uteri after Picrosirius red staining to show collagen fiber deposition (red arrows) in F1 (**A**) and F2 (**B**) female. Scale bars: 100 μm. Dashed black lines delineated the endometrium (ES) and myometrium (M).

Picrosirius red staining revealed a markedly higher collagen deposition in the endometrium of F1-D1 and F1-D2 (Figure 5A) and F2 (Figure 5B) females compared with their controls. Particularly, in the uterus of F1-D1, F1-D2, and F2 D1xC, D2xC, and D2xD2 (exposed father) females, endometrium and gland nests characterized by multiple uterine glands were surrounded by collagen-producing stromal cells (red arrows). This marked collagen accumulation might result in periglandular fibrosis. 

These results indicate that the myofibroblast population and collagen deposition were increased in the F1 and F2 endometrium, suggesting an association between NSAID and EE2 exposure and adenomyosis and potentially fibrosis.

### 2.5. Increased Proliferation of the Glandular Epithelium

**Figure 6:**
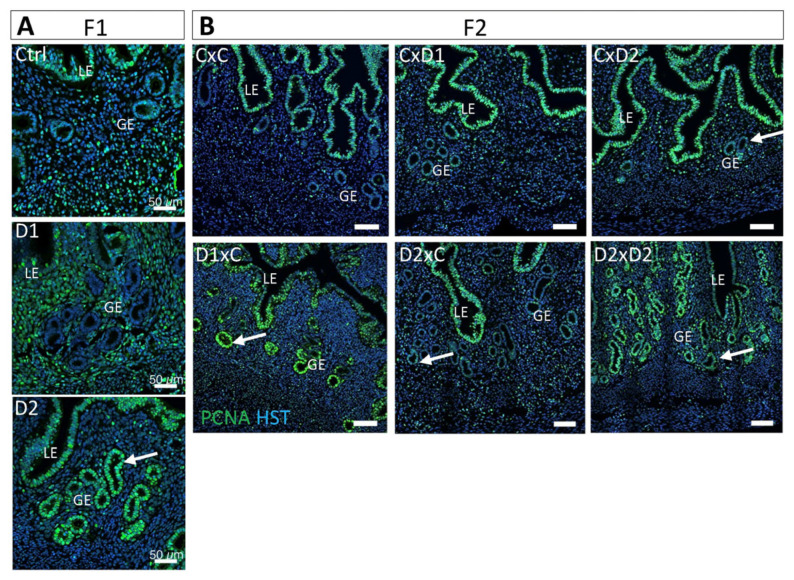
Exposure to the NSAID-EE2 mixture increases cell proliferation in the glandular epithelium. Representative immunofluorescence images of PCNA expression (green; cell proliferation marker) in 8-week-old F1 (**A**) and F2 (**B**) uteri. Hoechst dye (HST, in blue) highlights nuclei. Scale bars: 50 μm. LE: luminal epithelium; GE: glandular epithelium. White arrows, proliferating glandular epithelium cells.

Cell proliferation was investigated by IF with an anti-PCNA antibody (proliferation marker). Proliferation of glandular epithelial cells was observed in the uterus of F1-D2 (Figure 6A) and F2 D1xC, D2xC, and D2xD2 females (Figure 6B), but not in control and F1-D1 females. Glandular cell proliferation could be due to the altered tissue organization in the surrounding stroma that affects the epithelial–stromal crosstalk. 

### 2.6. Altered Localization of Cx43 Expression in the Glandular Epithelium

**Figure 7:**
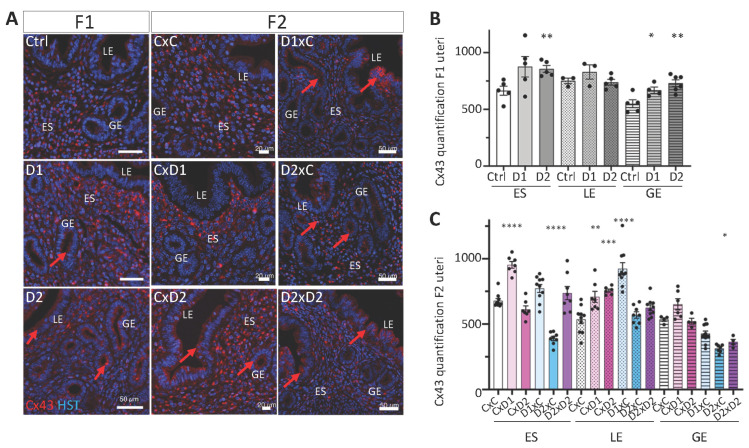
Cx43 (gap junction protein) expression and organization. (**A**) Paraffin-embedded uterus tissue sections of F1 and F2 females were stained with an anti-Cx43 antibody (red); nuclei were counterstained with Hoechst (HST; blue). Scale bars: 50 μm (F1 and F2 D1xC, D2xC and D2xD2) or 20 μm (F2 CxC, CxD1 and CxD2). ES: endometrial stroma; LE: luminal epithelium; GE: glandular epithelium. Red arrows indicate abnormal Cx43 expression in LE and GE. (**B**,**C**) Staining intensities of Cx43 were determined in *n* = 2–3 sections per uterus (*n* = 3–4 per group) (*n* = 4–10 ROIs per section) of ES (1500 μm^2^), LE (1000 μm^2^) and GE (700 μm^2^) (OMERO software). Data are the mean ± SEM, **** *p* < 0.001; *** *p* < 0.005; ** *p* < 0.01; * *p* < 0.05.

In the uterus, Cx43 is only expressed in the stromal compartment and plays significant roles in implantation and decidualization [13]. IF analysis showed Cx43 expression mainly in the endometrial stroma (ES) of the uterus from F1 and F2 controls, whereas luminal and glandular epithelial (LE and GE, respectively) cells were Cx43-negative (Figure 7A). Cx43 expression in the endometrial stroma (ES) was significantly increased in F1-D2 females, compared with controls (Figure 7B, *p* = 0.0049), and aberrant Cx43 expression was observed at the apical part of glandular epithelial (GE) cells (Figure 7A,B, *p* = 0.0373 (D1) and *p* = 0.0031 (D2), respectively). Like in F1 females, Cx43 expression was significantly increased in the endometrial stroma (ES) of F2 CxD1 (*p* < 0.0001) but decreased in F2 D2xC females (*p* < 0.0001), whereas it tended to increase non-significantly in F2 D1xC and D2xD2 females (Figure 7C). Cx43 expression was significantly increased in luminal epithelial (LE) cells in some F2 females (Figure 7A,C; CxD1 *p* = 0.0096; CxD2 *p* = 0.0003 and D1xC *p* < 0.0001). These results are in line with the aberrant epithelial distribution of Cx43 in ectopic endometriotic glands within the myometrium in cows with adenomyosis [14]. 

## 3. Discussion

These results indicated that exposure to NSAID and EE2 mixtures promotes the development of abnormal histological features and the formation of adenomyosis lesions (i.e., invasion of the inner layer of the myometrium by endometrial and stromal cells) in the uterus of exposed 8-week-old F1 females. These adverse effects were also observed in the uterus of F2 females, obtained mainly from mating combinations with an exposed father. Indeed, the uterus of F2 D1xC, F2 D2xC, and F D2xD2 females displayed similar phenotypes as the uterus of F1 females, often with more severe alterations of the luminal and glandular epithelia. Disease severity did not increase with age, and adenomyosis lesions did not regress after exposure end and up to 8 months of age, suggesting that they were caused by exposure during the embryonic [56] and postnatal [57] periods of uterine development. 

The toxicological data for each drug available in the literature suggest that the effects described in this study could be attributed to the exposure of both NSAIDs and EE2. In rodents, NSAIDs are considered antiandrogen molecules [43] and they inhibit the cyclooxygenase pathway, reducing the production of prostaglandins that are important mediators of uterine reproductive function [58,59]. Particularly, NSAIDs might inhibit the secretion of prostaglandin (PG) D2, which protects the endometrium against adenomyosis development through inhibition of PGE2 secretion [26]. As mixtures of many different chemicals are ubiquitously detected in effluents and surface water, risk assessment using NSAID and EE2 mixtures provides realistic toxicological data because both molecules may act simultaneously, leading to synergic dose addition and independent effects. This study showed that exposure to NSAIDs, such as IBU and DCF, and to EE2, even at environmentally relevant doses, has adverse effects on the endometrium. Therefore, these molecules might be added to the list of EDCs, such as BPA [60] and other persistent pollutants (phthalates, paraben) [61,62], that have endocrine disrupting activity and increase the risk of human endometriosis or adenomyosis.

Our results agree with those reported by Newbold et al. [35,63], who showed that neonatal treatment with BPA and DES induces alterations in the adult rat uterus. Similarly, tamoxifen administration in neonatal mice leads to early onset of adenomyosis after 40–90 days of treatment [15,16], and hypertrophy of all uterine layers at 6 weeks of age [17]. Female mice/rats exposed to BPA or DES also exhibited altered uterine luminal and glandular cell proliferation [36,64,65]. Uterine epithelium abnormalities, such as decreased epithelium height and increased number of glands, were also observed in mice treated with BPA analogs, bisphenol B and AF [66], and bisphenol S [67]. Ectopic invasion into uterine glands, adenomyosis, and adenocarcinoma spectrum lesions were observed in the uterus of aged mice exposed to EE2 prenatally and/or after sexual maturity [37]. In utero exposure to dioxin, another environmental toxicant that affects estrogenic signaling, promoted adenomyosis of the uterus, resulting in subfertility of the exposed mice [34]. Similarly, antiandrogen EDCs, such as tributyltin [40], and phthalates at environmentally relevant mixtures during prenatal stages [41] led to multigenerational and transgenerational effects on uterine cell morphology and functions in mice. Also, exposure to phthalates and environmental phenols, such as parabens, has been associated with gynecological disorders in women of reproductive age [68]. 

However, in this study, no significant modification of steroid hormone (E2 and P4) production was detected, probably due to the relatively low number of animals in each group and the high result variability. Furthermore, steroid hormone production is regulated by complex pathways that involve steroidogenic cells, paracrine regulation, feedback regulation with gonadotropins, and the balance between production and metabolism [69]. We previously showed that expression of steroidogenic enzymes contributing to E2 synthesis or metabolism was modified in the absence of PGD2 [26]. The differential expression of activating vs. inhibiting steroidogenic genes might vary from group to group, and the effect of NSAIDs and EE2 might be different, explaining why E2 availability was not significantly modified. Developmental exposure to xenoestrogen compounds, such as EE2 and BPA, alters the expression of steroid-sensitive genes that might lead to dysregulation of hormone signaling pathways [65] and, consequently, affect the uterine histology and uterine response to E2 and P4 in adulthood. 

Tissue injury and repair (TIR) is the most widely accepted molecular mechanisms involved in the pathophysiology of adenomyosis. Thus, understanding the complex microenvironment of the lesions and, in particular, the inflammatory, angiogenic, and endocrine signals are some priorities in subsequent research. Studies have shown that adenomyotic lesions microenvironment is the outcome of the interaction between immune cells and stromal and epithelial compartments. Dysregulation of proinflammatory cytokines (TNF-alpha, IL6,…) or IL24 has been demonstrated to play an important role in the pathophysiology of eutopic and ectopic endometriosis [70]. Moreover, alterations in local hormonal balance have been shown to create a positive feedback activation mechanism with inflammation, resulting in chemokines production and ECM modifications [71]. These microenvironment modifications could be initiated or enhanced by NSAIDs or EE2 interference.

Cx43 belongs to the connexin and gap junction family that has essential roles in the physiology of female reproductive organs [13]. This family is necessary for endometrial decidual differentiation and plays a role in implantation [13]. Upon exposure to NSAIDs and EE2, Cx43 was aberrantly expressed at the apical part of the luminal and glandular epithelial cells of F1 and F2 uteri. Similarly, aberrant epithelial distribution of Cx43 was reported in ectopic endometriotic glands of adenomyosis lesions in cows [14]. This strong Cx43 expression was correlated with high serum E2 and low P4 concentrations [14]. Cx43 staining was reduced in eutopic biopsies of women with endometriosis. Moreover, dispersed Cx43-positive cells were observed in the epithelium, while Cx43 was predominantly localized in the endometrial stroma in normal uteri [72]. Cx43 was upregulated in the luminal epithelium and endometrial stroma compartments of the uterus from DES-exposed female hamsters [64]. The connection between fibrosis and alterations in ECM signaling in uterine fibroids is well established [11]. The increased collagen deposition in the endometrial stroma of F1-D2 and most F2 females indicated a modified tissue following exposure. This might affect cell–cell communication and favor gland migration to the myometrium intergenerationally. Similarly, long-term exposure of young adult mice to low BPA doses increased gland nest density and periglandular collagen accumulation, contributing to increased fibrosis [73]. Perinatal administration of BPA also altered the expression of tight junction proteins, such as claudin-4 and -7, and ZO-1, in the rat neonatal uterus, leading later to a reduced number of implantation sites in pregnant adult females [74]. Furthermore, prenatal exposure of mice to phthalates increased collagen deposition in the endometrium and myometrium, which was associated with the presence of multilayered luminal epithelium in multiple generations [41].

Thus, the aberrant presence of myofibroblasts in the endometrium-junctional zone and extracellular matrix dysregulation might contribute to the development of abnormal histological features and adenomyosis in F1 and F2 uteri that might alter the uterus physiology. Indeed, uterine epithelial cells, endometrial stromal cells, and endometrial glands play critical roles in establishing and maintaining pregnancy-related cell proliferation and embryo survival [75,76]. Also, endometrial atrophy has been associated with adverse pregnancy complications [77]. Thus, our results might explain the subfertility of F1 and F2 females after 4–5 months of age [53]. 

However, our study suffers from a number of limitations. The most important is the lack of study of possible molecular mechanisms and target genes. Further studies will be needed to clarify the different signaling pathways involved in the development of adenomyosis. Secondly, the transgenerational effects observed in our study involve mechanisms that also need to be clarified. Finally, our data highlight the effect that cocktails of EDCs can have on the development of adenomyosis. The authorities would, therefore, be well advised to take steps to reduce more drastically the number and concentration of these substances in drinking water.

## 4. Material and Methods

### 4.1. Animals and Study Design

**Figure 8:**
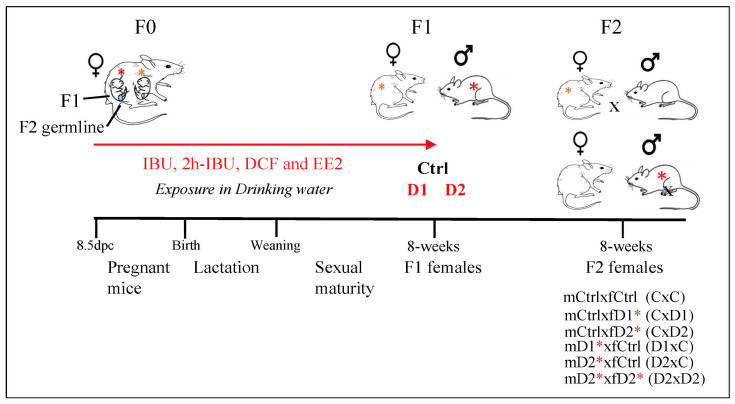
Experimental design: exposure to two doses of the same mixture, Dose 1 (D1) and Dose 2 (D2), that contains ibuprofen (IBU)/2hydroxyIbuprofen (2hIBU), diclofenac (DCF) and 17α-ethinylestradiol (EE2) in drinking water of pregnant mice, from 8.5 dpc until sacrifice (8 weeks of age), to generate F1-D1 and F1-D2 animals (exposed males and females are highlighted by red and orange asterisks, respectively). F1 males (m) or females (f) were mated with control males (mCtrl) or females (fCtrl) (*n* = 6 groups: mCtrlxfC, mCtrlxfD1, mCtrlxfD2, mD1xfCtrl, mD2xfCtrl, and mD2xfD2) (these mating combinations were simplified to CxC, CxD1, CxD2, D1xC, D2xC, and D2xD2, respectively). Red asterisks highlight the F1 animals exposed to the mixture.

To generate F1 animals, CD-1 mice (F0) (Charles River Laboratories, Les Oncins, Saint Germain Nuelles, France) were mated, and pregnant F0 females were divided into three groups: control (no exposure, *n* = 6), mixture dose 1 (D1, *n* = 6), and mixture dose 2 (D2, *n* = 7) (Figure 8). Pregnant F0 females were exposed or not (control) to one of the two doses of the IBU, 2h-IBU, DCF, and EE2 mixture from 8.5 days post coitum (dpc) to birth, and then their F1 progeny was exposed to the same mixture up to adult age (8 weeks). This exposure time covered the main steps of migration, proliferation, and differentiation/maturation of germ cells and somatic cell lineages from the early embryonic stages until sexual maturity. The two doses in the drinking water were calculated based on their mean concentrations (D1: 5, 40, 10, and 1 ng/L, respectively) and maximum concentrations (D2: 50, 100, 50, and 20 ng/L, respectively) found in environmental drinking water samples (ANSES referral 2013-SA-0081) [78], as previously reported [52]. The control group was exposed to 0.001% ethanol (vehicle). Then, F1 exposed males (mD1, mD2) or females (fD1, fD2) were mated with control males (mCtrl) or females (fCtrl) or with D2 males (mD2) or females (fD2) to obtain F2 females (mCxfC, mCxfD1, mCxfD2, mD1xfC, mD2xfC, and mD2xfD2; *n* = 5–7 matings/group) (Figure 8). These mating combinations were simplified in the manuscript and named CxC, CxD1, CxD2, D1xC, D2xC, and D2xD2, respectively. For this study, animals were reared according to the protocols described previously [52,53]. All animal experiments were carried out in accordance with procedures approved by the Réseau des Animaleries de Montpellier (approval number 34-366 for B.B.-B. animal experimentation) and by the French regional ethics committee. Mice were euthanized by cervical dislocation in accordance with the recommendations for animal experiments. The 3R rule was respected throughout this project.

In each litter (F1 and F2), at least two 8-week-old females were sampled. Similarly, at the end of the fertility study [53], 8-month-old F1 females (*n* = 9 to 12) were sampled. For each uterus, one uterine horn was fixed in 4% paraformaldehyde (in PBS) and embedded in paraffin for histological analysis, and the other horn was immediately frozen in dry ice and conserved at −80 °C for hormone assays.

### 4.2. Uterus Histological Analysis

F1 and F2 uterus samples embedded in paraffin were cut in 3-μm sections and were stained with hematoxylin–eosin (HE) using standard protocols to examine the tissue structure and morphology (RHEM Histology platform BioCampus, Montpellier, France). Nascent adenomyotic lesions were identified either by a disordered endometrium-myometrium interface without endometrial glands included in the myometrium or by glands/stroma beginning to penetrate the myometrium. Paraffin-embedded uterus sections were stained with Picrosirius red stain to detect connective tissue and collagen (signs of fibrosis) [79]. Stained sections were scanned with a Nanozoomer Hamamatsu device (Hamamatsu Photonics, Tokyo, Japan) and analyzed with the Nanozoomer Digital Pathology (NDPview2 U12388-01) software (Hamamatsu Photonics). To assess the incidence of epithelial or glandular abnormalities, the number of mice with at least one abnormality of the chosen type was divided by the total number of animals per group. 

### 4.3. Immunofluorescence Analysis and Staining Quantification

For immunofluorescence (IF) analysis, tissue sections were processed as previously described [52,80] and incubated with the following primary antibodies: polyclonal rabbit anti-FOXL2 (homemade [81], 1/300), anti-α-SMA (Abcam ab124964, 1/500, Cambridge, UK), anti-Cx43 (Sigma Aldrich C6219, 1/400, St. Louis, MO, USA), and monoclonal mouse anti-PCNA (Sigma Aldrich P8825, 1/500), anti-pan-cytokeratin (Thermo Fisher MA5-13156, 1/400, Waltham, MA, USA). Sections were then incubated with donkey or goat secondary antibodies conjugated with Alexa Fluor 555, 488, or 649 (Thermo Fisher, 1/1000), followed by Hoechst staining. IF images were captured with a Zeiss AxioImager apotome microscope (Carl Zeiss Microscopy, Jena, Germany) at the IGH Imaging facility (BioCampus) and processed with the OMERO software (OMERO web 5.5.1., University of Dundee and Open Microscopy Environment, Dundee, UK). The intensity of α-SMA staining was determined in regions of interest (ROIs) (*n* = 4–12 ROIs per section) of a constant surface (30 mm^2^) at the endometrial stroma-junctional zone (2–3 sections of each uterus, *n* = 4–7 per group). Cx43 staining intensity was determined in ROIs (*n* = 4–10 ROIs per section, *n* = 3 per group) in the endometrial stroma (1500 μm^2^), luminal epithelium (1000 μm^2^), and glandular epithelium (700 μm^2^) (OMERO software). Data were analyzed with GraphPrism 7.

### 4.4. Hormone Dosages

Steroids were extracted (twice) from uterus tissue samples (10–40 mg) with diethyl ether. After evaporation, extracts were resuspended in EIA buffer (Cayman Chemical, Ann Arbor, MI, USA). Progesterone (P4) and 17β-estradiol (E2) were quantified using Enzyme-linked immunosorbent assays (ELISA) (Cayman Chemicals 582601 and 501890, respectively) according to the manufacturer’s instructions. The analytical sensitivity of these assays was 10 pg/mL for P4 (assay range 7.8–1000 pg/mL) and 20 pg/mL for E2 (assay range 0.61–10,000 pg/mL). Steroid levels were normalized to the uterus sample weight and expressed in pg/mL/mg tissue.

### 4.5. Statistical Analysis

Statistical analyses were performed with GraphPad Prism 7. One-way ANOVA with Tukey’s post hoc test was used for multiple comparisons. All values are represented as the mean ± SEM of several independent experiments (*n* > 3); *p* < 0.05 was considered significant. 

## 5. Conclusions

This study showed that chronic exposure to environment-relevant doses of NSAID (IBU and DCF) and EE2 mixtures in drinking water induced abnormal histological features and adenomyosis lesions in the uterus of exposed F1 females and their F2 offspring. Describing the uterus histopathology following exposure to environmentally relevant doses of NSAIDs and EE2 will provide more general insights into adenomyosis/endometriosis pathogenesis and EDC-associated female reproductive disorders, such as infertility, endometrial hyperplasia, recurrent miscarriage, and even endometrial cancer. Future studies must investigate the mechanisms of these intergenerational effects that might increase reproductive health risks in multiple generations.

## Data Availability

Data is contained within the article.
